# Reverse Takotsubo Cardiomyopathy in a Woman With Clostridioides difficile Colitis and a Sepsis-Like Syndrome: A Case Study

**DOI:** 10.7759/cureus.97512

**Published:** 2025-11-22

**Authors:** Naomi R Khanna, Adil Pervaiz

**Affiliations:** 1 Cardiology, Advanced Cardiology, Hackettstown, USA; 2 Biomedical Engineering, Columbia University, New York, USA; 3 Cardiology, Morristown Medical Center, Cedar Knolls, USA

**Keywords:** apical hyperkinesis, basal akinesis, catecholamine-mediated cardiac dysfunction, echocardiography findings, recovery of left ventricular function, reverse takotsubo cardiomyopathy (rttc), stress-induced cardiomyopathy

## Abstract

In this report, we present a case of reverse Takotsubo cardiomyopathy (rTTC) in an elderly female, 74 years old, with Clostridioides difficile colitis and sepsis-like features complicated by hypotension and arrhythmias. The diagnosis was eventually established through echocardiography and cardiac catheterization. Supportive therapy favored recovery after diagnosis. This case of rTTC highlights a serious, although rare, cardiac condition that emphasizes the importance of workup and recognition to support optimal management and recovery and underscores the need to keep stress-induced cardiomyopathies in mind in critically ill patients.

## Introduction

Takotsubo cardiomyopathy (TTC) is a cardiac condition marked by apical akinesis with basal hyperkinesis, typically due to emotional or physical stress and disproportionately seen in postmenopausal women. TTC is characterized by transient regional systolic dysfunction of the left ventricle without obstructive coronary artery disease [1]. In its typical form, there is apical akinesis and ballooning with basal hyperkinesis, giving the heart the appearance of a “Takotsubo,” which refers to a Japanese pot traditionally used to trap octopuses [2]. Reverse Takotsubo cardiomyopathy (rTTC) is an uncommon presentation in which there is the reverse pattern of basal akinesis and preserved or hyperkinetic apical function [2]. While the causes of rTTC and TTC are unclear, it is hypothesized that elevated catecholamines, coronary spasm, microvascular dysfunction, and inflammation may play a role [1]. rTTC is typically treated using conservative medical management with beta-blockers and angiotensin-converting enzyme (ACE) inhibitors or angiotensin receptor blockers [3].

This case report showcases the presentation of rTTC in a 74-year-old woman with Clostridioides difficile colitis and a sepsis-like syndrome. Septic and toxin-mediated stress are known to cause a catecholamine surge and could serve as triggers that result in myocardial depression. Although any stressor can cause TTC, rTTC arising from C. difficile colitis is exceptionally rare, making this case novel.

## Case presentation

This case presents a 74-year-old woman with a medical history of anxiety and chronic lower back pain. She was admitted to the hospital with generalized weakness and dizziness. During her stay, she began complaining of lower abdominal pain and agitation. Her stool tested positive for C. difficile. Over the course of a few hours, her blood pressure dropped transiently, and she was transferred to the ICU with a presumptive diagnosis of sepsis.

In the ICU, the patient had several self-terminating runs of atrial fibrillation and wide complex tachycardia. Her N-terminal pro-brain natriuretic peptide (NT-proBNP) level was 29,600 pg/mL (normal for her age <900), and her troponin I level was 3,300 ng/L (normal <50). However, her troponin peak was flat, and her EKG showed no new ST changes, with a QT interval of 414 ms.

**Figure 1:**
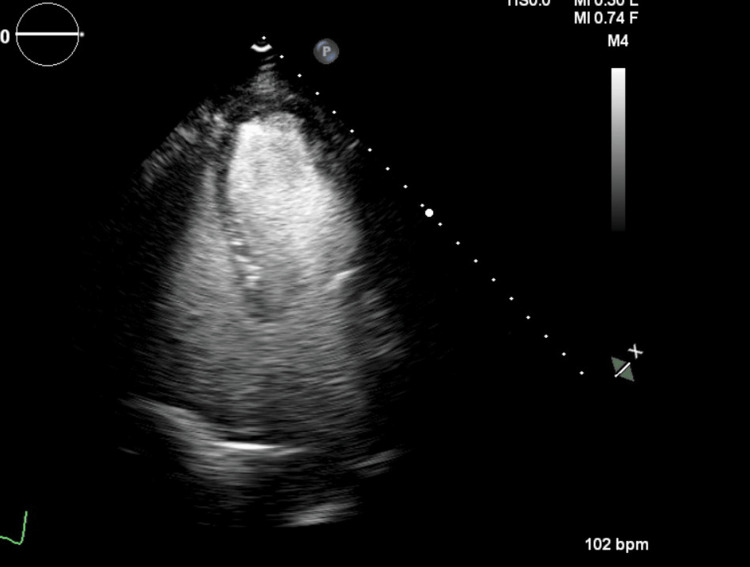
Transthoracic echocardiogram during diastole

**Figure 2:**
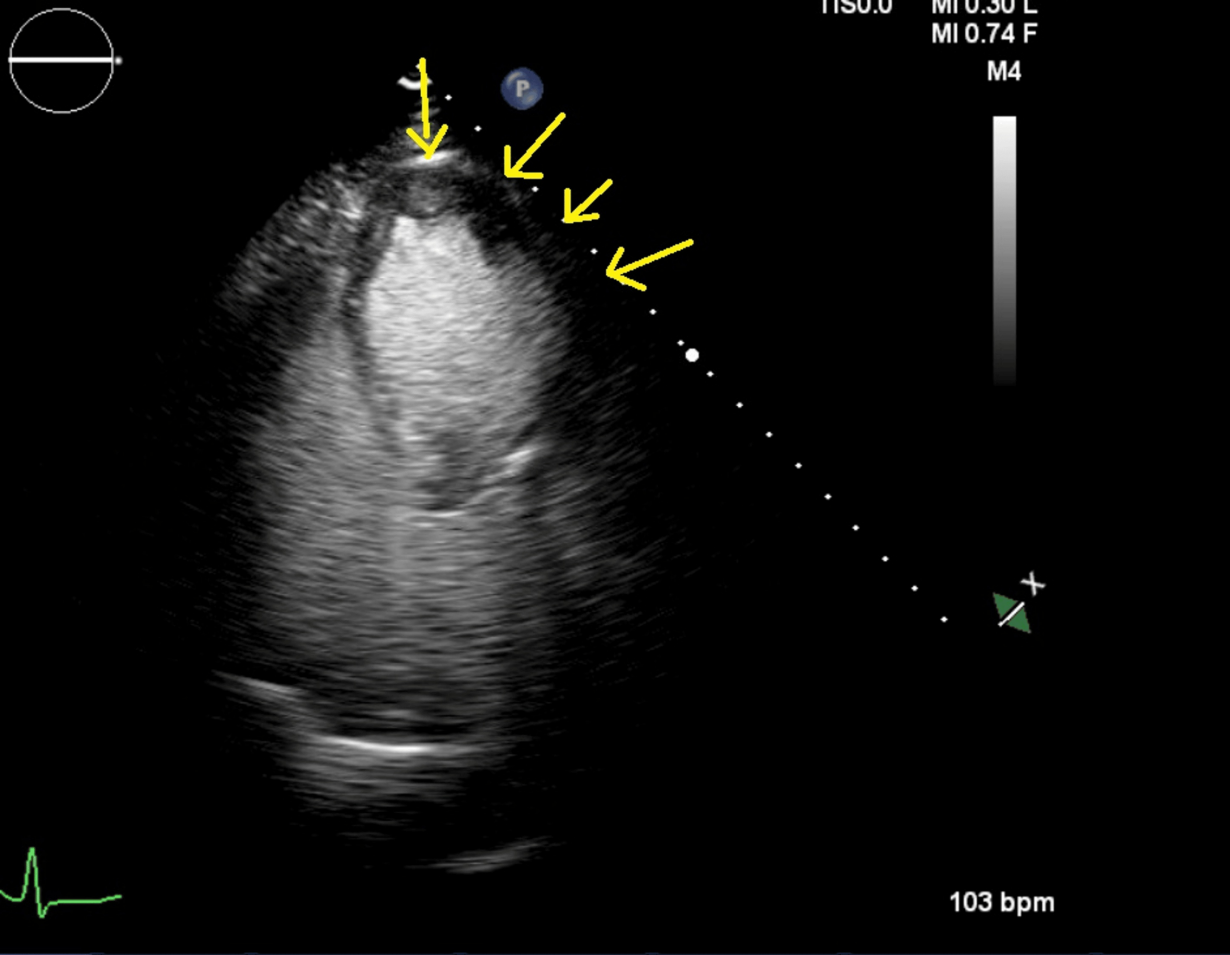
Transthoracic echocardiogram in systole demonstrating akinesis of the basal and midventricular segments with preserved apical contraction (marked by arrows)

An echocardiogram revealed an ejection fraction (left ventricular ejection fraction (LVEF)) of 20-30%. There was akinesis of the midventricular and basal segments, and apical contractility was preserved (Figure 1 and Figure 2).

The patient was transferred to a tertiary care center. Coronary angiography was essentially normal, but she had an elevated pulmonary artery (PA) wedge pressure (26 mmHg) and a severely reduced cardiac index. She was briefly maintained on inotropic support (milrinone infusion) with hemodynamic monitoring using a PA catheter. Her hemodynamics improved over the next 48 hours, her inotropic support was stopped, and the PA catheter was removed. Metoprolol, losartan, and spironolactone were initiated, and she was downgraded to the floor. She was discharged home on day 11 on a small dose of beta-blockers and ACE inhibitors.

**Figure 3:**
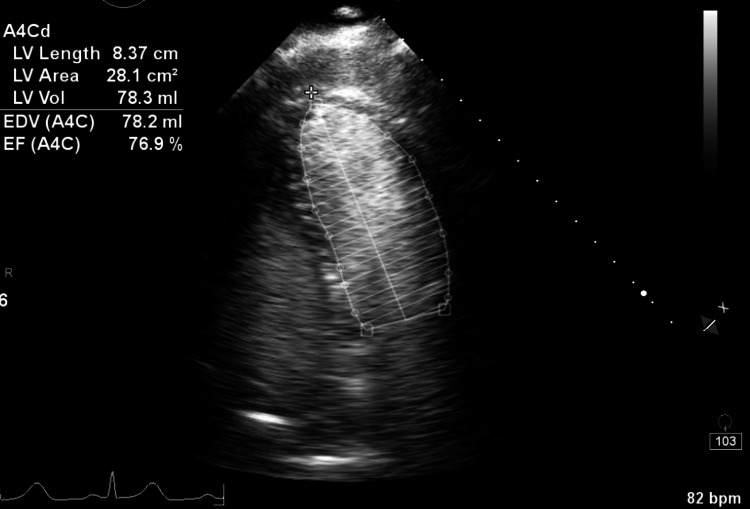
Recovery of LVEF LVEF, left ventricular ejection fraction

At discharge, her LVEF remained diminished in the 30-35% range. Her NT-proBNP level normalized to 179 pg/mL. Follow-up echocardiogram showed full recovery of systolic function in the previously akinetic segments and a normal LVEF of greater than 70% (Figure 3).

## Discussion

TTC is characterized by transient regional systolic dysfunction of the left ventricle in the absence of obstructive coronary artery disease [1]. Since its initial description, variant or atypical forms of the disease with different patterns of contractile dysfunction have been reported. The most common variant is the reverse type (rTTC), characterized by a hyperdynamic apex and akinesis of the basal segments of the left ventricle [4]. This may be explained by regional variations in adrenergic sensitivity or innervation of the myocardium among individuals [4].

TTC is seen in approximately 2% of patients presenting with suspected acute coronary syndrome, and rTTC accounts for roughly 2.2% of those cases, though the reported incidence varies across studies. It is seen more in younger patients [3-5].

The exact mechanism of TTC and rTTC remains unknown, but several hypotheses have been proposed, including sympathetic overdrive with elevated catecholamines, coronary spasm, microvascular dysfunction, low estrogen levels, inflammation, and impaired myocardial fatty acid metabolism [1].

The clinical presentation of reverse rTTC, like TTC, often includes symptoms such as chest pain, dyspnea, abdominal pain, diaphoresis, syncope, or indigestion. Patients may also show signs of congestive heart failure or hypotension [2]. However, in critically ill individuals, symptoms can be nonspecific or masked by other systemic conditions such as sepsis. Both TTC and rTTC are defined as acute cardiac events characterized by the following: (I) transient abnormal wall motion in the form of hypokinesis, akinesis, or dyskinesis of the left ventricular basal segments, with regional involvement extending beyond a single epicardial vascular territory; (II) absence of obstructive coronary artery disease or angiographic evidence of acute plaque rupture; (III) new ECG abnormalities or elevated cardiac troponin levels; and (IV) exclusion of myocarditis or pheochromocytoma [2,6].

It is the echocardiographic pattern of a hyperdynamic apex and akinesis of the basal segments of the left ventricle that distinguishes rTTC from the more commonly seen TTC, where the apex is akinetic and the base is hyperkinetic.

Treatment for rTTC typically involves ACE inhibitors and beta-blockers. Furthermore, in cases with significant ventricular dysfunction, pharmacologic and hemodynamic support can rapidly restore left ventricular function and improve survival [2].

The prognosis of rTTC with regard to recurrence and mortality is similar to that of TTC [2].

Recent observational data suggest that rates of cardiogenic shock and mortality in TTC are comparable to those seen in patients with acute coronary syndrome, with an overall mortality of 4.1% reported in the Takotsubo Registry study [1].

## Conclusions

This case describes reverse TTC in an elderly woman with C. difficile colitis and a sepsis-like syndrome. The diagnosis of cardiac dysfunction was suspected due to hypotension and transient arrhythmias. rTTC as the cause of the cardiac dysfunction was confirmed through echocardiography and cardiac catheterization. Her cardiac function improved rapidly with supportive care and temporary inotropic therapy. As seen in most patients with rTTC, she made a complete recovery.

This case underscores the importance of considering stress-induced cardiomyopathies such as rTTC in critically ill patients where there is septic or toxin-mediated stress, even when the presentation is not typical of acute coronary syndromes.
